# Dysbiosis and compositional alterations with aging in the gut microbiota of patients with heart failure

**DOI:** 10.1371/journal.pone.0174099

**Published:** 2017-03-22

**Authors:** Takehiro Kamo, Hiroshi Akazawa, Wataru Suda, Akiko Saga-Kamo, Yu Shimizu, Hiroki Yagi, Qing Liu, Seitaro Nomura, Atsuhiko T. Naito, Norifumi Takeda, Mutsuo Harada, Haruhiro Toko, Hidetoshi Kumagai, Yuichi Ikeda, Eiki Takimoto, Jun-ichi Suzuki, Kenya Honda, Hidetoshi Morita, Masahira Hattori, Issei Komuro

**Affiliations:** 1 Department of Cardiovascular Medicine, Graduate School of Medicine, The University of Tokyo, Tokyo, Japan; 2 Graduate School of Frontier Sciences, The University of Tokyo, Kashiwa, Japan; 3 Department of Microbiology and Immunology, Keio University School of Medicine, Tokyo, Japan; 4 Department of Advanced Clinical Science and Therapeutics, Graduate School of Medicine, The University of Tokyo, Tokyo, Japan; 5 RIKEN Center for Integrative Medical Sciences, Yokohama, Japan; 6 Graduate School of Environmental and Life Science, Okayama University, Okayama, Japan; 7 Graduate School of Advanced Science and Engineering, Waseda University, Tokyo, Japan; Nagoya University, JAPAN

## Abstract

Emerging evidence has suggested a potential impact of gut microbiota on the pathophysiology of heart failure (HF). However, it is still unknown whether HF is associated with dysbiosis in gut microbiota. We investigated the composition of gut microbiota in patients with HF to elucidate whether gut microbial dysbiosis is associated with HF. We performed 16S ribosomal RNA gene sequencing of fecal samples obtained from 12 HF patients and 12 age-matched healthy control (HC) subjects, and analyzed the differences in gut microbiota. We further compared the composition of gut microbiota of 12 HF patients younger than 60 years of age with that of 10 HF patients 60 years of age or older. The composition of gut microbial communities of HF patients was distinct from that of HC subjects in both unweighted and weighted UniFrac analyses. *Eubacterium rectale* and *Dorea longicatena* were less abundant in the gut microbiota of HF patients than in that of HC subjects. Compared to younger HF patients, older HF patients had diminished proportions of Bacteroidetes and larger quantities of Proteobacteria. The genus *Faecalibacterium* was depleted, while *Lactobacillus* was enriched in the gut microbiota of older HF patients. These results suggest that patients with HF harbor significantly altered gut microbiota, which varies further according to age. New concept of heart-gut axis has a great potential for breakthroughs in the development of novel diagnostic and therapeutic approach for HF.

## Introduction

In the human gut, there are more than 10^14^ bacterial cells, which exceed the number of human cells in the body. Their combined genomes contain millions of genes, which are hundred times the number of human genes. These large quantities of gene products complement host metabolism and facilitate the development of host immune system [1, 2]. In line with the crucial link between gut microbiota and the maintenance of host health, there is growing evidence that altered composition of gut microbiota, known as dysbiosis, contributes to the pathogenesis of host diseases [3]. Numerous experiments with fecal microbiota transplantation to germ-free animals have suggested that gut microbiota can initiate and influence host diseases such as obesity-related diseases, liver diseases, inflammatory bowel diseases, and colorectal cancer [4].

In patients with heart failure (HF), the structure and function of the gut are altered as a consequence of microcirculatory disturbances [5, 6]. Impaired epithelial absorption may have detrimental effect on nutritional status of patients with HF, and disruption of epithelial barrier may lead to translocation of microbial products into systemic circulation, possibly aggravating HF by inducing systemic inflammatory responses [7–10]. Indeed, compared with healthy control (HC) subjects, patients with HF showed increases in the quantity of pathogenic bacteria in feces and the density of bacteria adhered to colon mucosa [5, 6], in association with an increase in intestinal permeability [5]. Gut microbe-derived metabolites such as indoxyl sulfate and trimethylamine N-oxide (TMAO) may also contribute to the pathogenesis of HF through undefined mechanisms [11–13]. Therapeutic management of HF through manipulating gut microbiota is under investigation in animal models. For example, oral administration of antibiotics or probiotics to rats has been reported to reduce myocardial infarct size in ischemia-reperfusion injury and to attenuate cardiac remodeling after myocardial infarction [14, 15]. These observations suggest a significant impact of gut microbiota on the pathophysiological process of HF. However, it is unclear whether dysbiosis in gut microbiota is associated with HF.

To address this issue, we analyzed the gut microbiome of HF patients and HC subjects using 16S ribosomal RNA (rRNA) gene sequencing. Our data revealed the presence of dysbiosis in the gut microbiota of patients with HF. Moreover, the gut microbiota composition of older HF patients differed from that of younger HF patients. Our studies provide new insights into the heart-gut axis in the pathophysiology of HF, and pave the way toward exploring the potential of manipulating gut microbiota as a future therapeutic strategy against HF.

## Materials & methods

### Study population

We recruited a total of 22 patients with HF (New York Heart Association functional class II to IV) who were hospitalized at the University of Tokyo Hospital. All patients were hospitalized for acute decompensated HF or acute exacerbation of chronic HF. These HF patients were classified into 2 groups according to age, those younger than 60 years of age (n = 12, aged 47.4 ± 2.8 years, 11 men and 1 woman) and those 60 years of age or older (n = 10, aged 73.8 ± 2.8 years, 7 men and 3 women). We excluded the patients with clinical signs of active infection, chronic inflammatory diseases, malignancy, renal failure requiring renal replacement therapy, or a history of gastrointestinal surgery. In addition, exclusion criteria included receiving antibiotic, probiotic, steroid, or immunosuppressive therapy during the previous 2 months. Twelve age-matched healthy volunteers (aged 41.4 ± 2.0 years, 9 men and 3 women) were recruited as HC subjects at Azabu University. Clinical characteristics of all subjects are listed in S1–S3 Tables. This study complies with the Declaration of Helsinki, and was approved by the Research Ethics Committee, Graduate School of Medicine and Faculty of Medicine, The University of Tokyo and the Human Research Ethics Committee of Azabu University. The written informed consent was obtained from all of the subjects.

### Fecal sample collection and bacterial DNA extraction

Fecal samples were freshly collected and transported to the laboratory under anaerobic condition in AnaeroPack (Mitsubishi Gas Chemical Company, Inc., Tokyo, Japan) at 4°C. The fecal samples were frozen by liquid nitrogen in phosphate-buffered saline containing 20% glycerol, and stored at -80°C until use. Bacterial DNA was extracted from the fecal samples by enzymatic lysis method using lysozyme (Sigma-Aldrich Co., St. Louis, Missouri) and achromopeptidase (Wako Pure Chemical Industries, Ltd., Osaka, Japan), as previously described [16, 17].

### Sequencing of 16S ribosomal RNA gene amplicons

Bacterial DNA from the fecal samples was amplified by PCR, as previously described [17]. Primers 27Fmod and 338R with adaptor sequences for 454 pyrosequencing were used to amplify the bacterial 16S rRNA gene V1-V2 region. PCR was run for 25 cycles, using Ex Taq polymerase (Takara Bio Inc., Kusatsu, Japan). PCR amplicons were purified by AMPure XP magnetic purification beads (Beckman Coulter, Inc, Brea, California) and quantified using the Quant-iT PicoGreen dsDNA Assay Kit (Thermo Fisher Scientific Inc., Waltham, Massachusetts). Equal amount of amplicons from each sample were sequenced with 454 GS FLX Titanium or 454 GS JUNIOR (Roche Applied Science, Indianapolis, Indiana) according to the manufacturer’s instructions.

### Data analysis

The previously established analysis pipeline was utilized for data analysis [17]. Filter-passed 3,000 reads, with an average quality score of 25 or higher, were randomly selected from the reads for each sample. The number of operational taxonomic units (OTUs) in each sample was calculated by clustering the 3,000 reads at a 96% identity threshold. The richness and diversity of microbial communities in each sample were evaluated by Chao1-estimated OTU number and Shannon index respectively. For taxonomic assignment, the read sequences were aligned against the 16S rRNA gene database constructed from RDP, CORE, and NCBI genome databases, and were assigned to taxonomic groups at a 96% identity threshold. Taxonomic groups with relative abundance in any subject above 0.1% were included in the analysis. UniFrac analysis was used to calculate phylogenetic tree-based distances between microbial communities of the individuals [18].

### Statistical analysis

Data are presented as mean ± SEM. The unpaired Student *t* test was used to evaluate the between-group differences. Values of p < 0.05 were considered statistically significant.

## Results

### Gut microbiota in patients with heart failure and healthy control subjects

**Fig 1:**
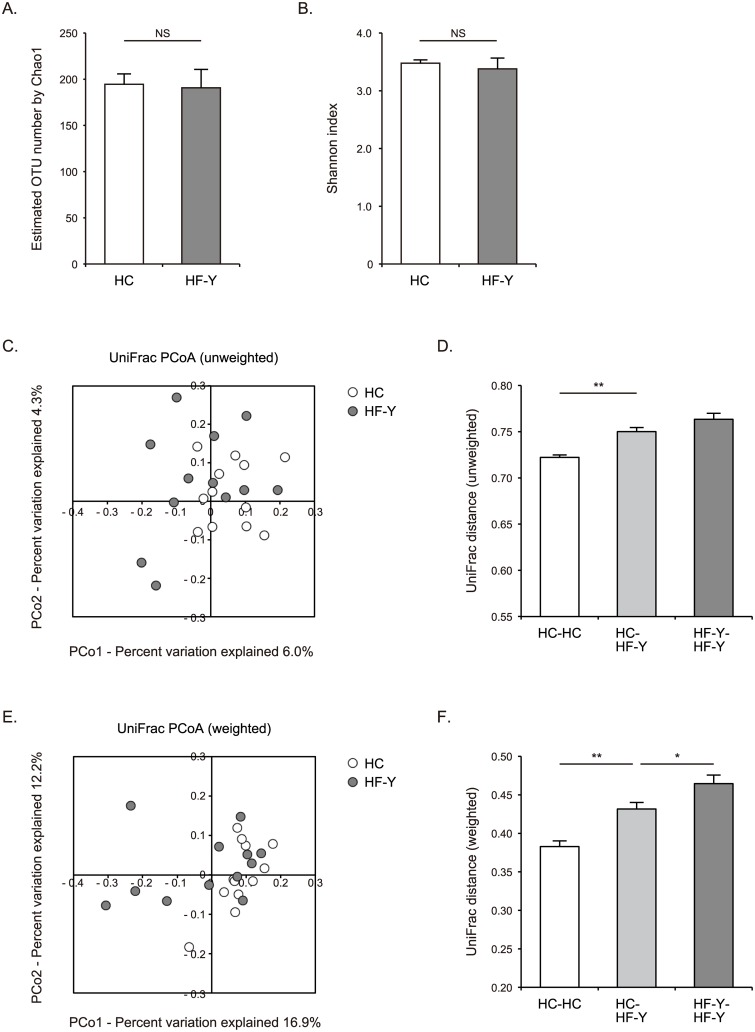
Richness, diversity, and UniFrac distances of gut microbiota in heart failure patients and healthy control subjects. Chao1-estimated operational taxonomic unit (OTU) number **(A)** and Shannon index **(B)** of gut microbiota samples obtained from younger heart failure (HF-Y) patients and healthy control (HC) subjects. Unweighted UniFrac analysis **(C, D)** and weighted UniFrac analysis **(E, F)** of gut microbiota samples obtained from HF-Y patients and HC subjects. Principal Coordinate Analysis (PCoA) of UniFrac distances between gut microbial communities of the individuals **(C, E)**, and UniFrac distances between gut microbial communities of the individuals within each group and between the two groups **(D, F)**. Data are presented as mean ± SEM. NS, not significant. * p < 0.05, ** p < 0.00001.

We performed 16S rRNA gene sequencing of fecal samples from 12 younger HF patients (younger than 60 years of age) and 12 age-matched HC subjects. Gut microbial richness in the given individual was measured by Chao1-estimated OTU number, and gut microbial diversity in the individual was evaluated by Shannon index. The richness and diversity of gut microbiota were similar between the samples from HF patients and HC subjects (Chao1-estimated OTU number: 191 ± 20 vs. 195 ± 12, Shannon index: 3.38 ± 0.19 vs. 3.48 ± 0.06) (Fig 1A and 1B). We next estimated the distances between fecal samples obtained from the individuals using UniFrac analysis [18, 19]. UniFrac distances between gut microbial communities of the individuals were visualized by a scatter plot created by Principal Coordinate Analysis (PCoA). Unweighted UniFrac is a qualitative measure that reflects inter-individual differences in the presence or absence of each taxon. Weighted UniFrac is a quantitative measure that reflects inter-individual differences in the relative abundance of each taxon. Mean unweighted and weighted UniFrac distances between gut microbiota of HF patients and HC subjects were 0.750 and 0.432, respectively, which were greater than inter-individual UniFrac distances in the gut microbiota of HC subjects (0.722 and 0.383, respectively, both p < 0.00001) (Fig 1C–1F). Accordingly, the composition of gut microbial communities of HF patients was distinct from that of HC subjects in both unweighted and weighted UniFrac analyses. These data also showed greater inter-individual diversity in the gut microbiota of HF patients compared to HC subjects (Fig 1C–1F).

**Fig 2:**
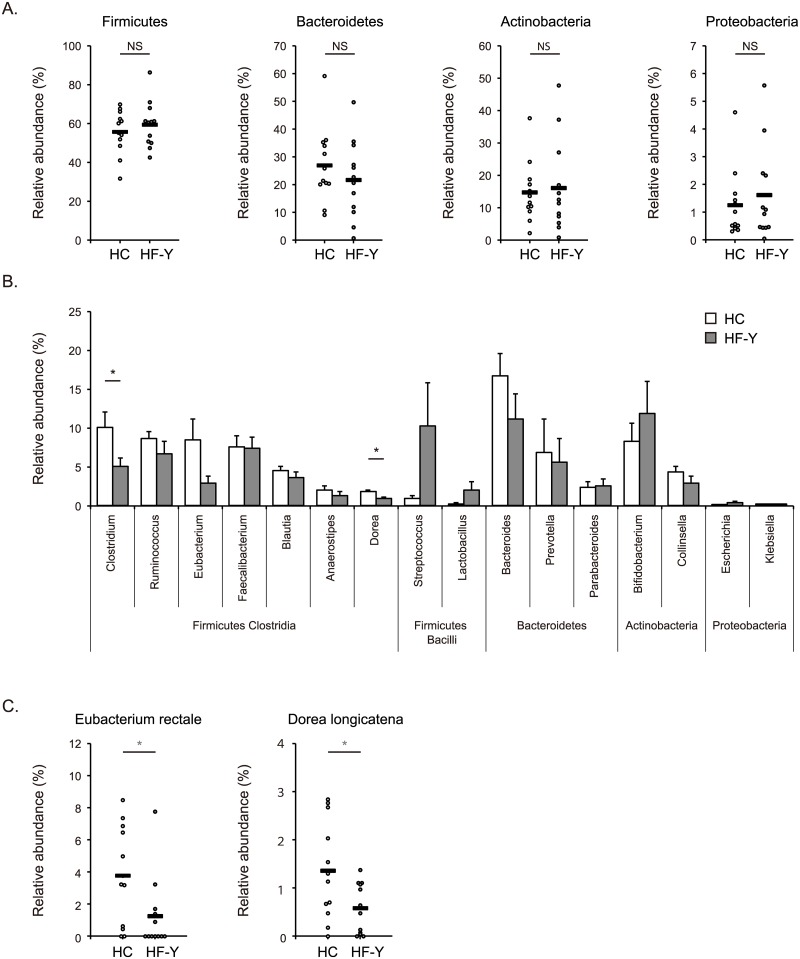
Abundances of taxa in gut microbiota of heart failure patients and healthy control subjects. Relative abundances of taxa in gut microbiota samples obtained from younger heart failure (HF-Y) patients and healthy control (HC) subjects. **(A)** Phylum level. **(B)** Genus level. **(C)** Species level. Data are presented as mean ± SEM. Horizontal bars indicate means. * p < 0.05. NS, not significant.

To investigate whether HF patients had significant changes in specific taxonomic groups of gut microbial communities, we analyzed the relative abundances of 16S rRNA reads assigned to each phylum, genus, or species. The majority of gut microbiota was dominated by the four phyla, Firmicutes, Bacteroidetes, Actinobacteria, and Proteobacteria. Significant differences were not observed between the samples from HC subjects and HF patients in terms of relative abundances of respective phyla (Firmicutes: 55.8 ± 3.2% vs. 59.4 ± 3.4%, Bacteroidetes: 27.0 ± 3.9% vs. 21.7 ± 4.1%, Actinobacteria: 14.7 ± 2.7% vs. 16.1 ± 4.1%, Proteobacteria: 1.3 ± 0.4% vs. 1.6 ± 0.5%) (Fig 2A). Taxonomic assignment performed at the genus level demonstrated that *Clostridium* and *Dorea* were less abundant in the gut microbiota of HF patients than in that of HC subjects (5.1 ± 1.1% vs. 10.1 ± 2.0%, p = 0.040, and 0.9 ± 0.2% vs. 1.8 ± 0.3%, p = 0.039, respectively) (Fig 2B). At the species level, *Eubacterium rectale* and *Dorea longicatena* were significantly reduced in the samples from HF patients compared to HC subjects (1.2 ± 0.7% vs. 3.8 ± 0.9%, p = 0.032, and 0.6 ± 0.2% vs. 1.4 ± 0.3%, p = 0.031, respectively) (Fig 2C).

### Gut microbiota in younger and older patients with heart failure

**Fig 3:**
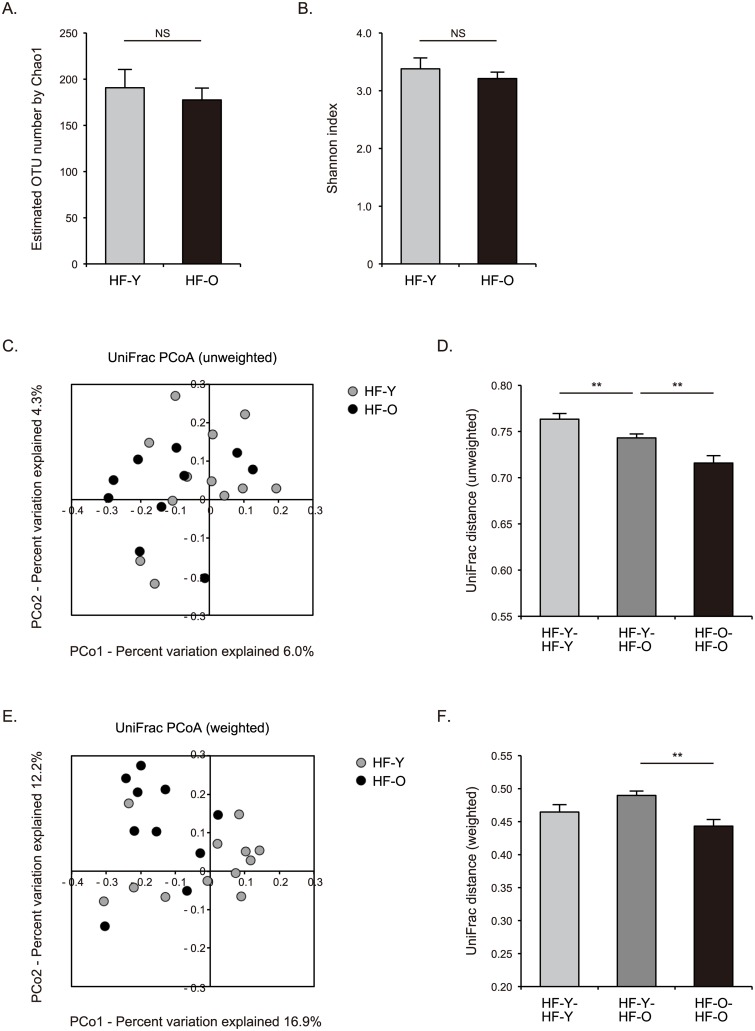
Richness, diversity, and UniFrac distances of gut microbiota in younger and older patients with heart failure. Chao1-estimated operational taxonomic unit (OTU) number **(A)** and Shannon index **(B)** of gut microbiota samples obtained from younger and older patients with heart failure (HF-Y and HF-O, respectively). Unweighted UniFrac analysis **(C, D)** and weighted UniFrac analysis **(E, F)** of gut microbiota samples obtained from HF-Y and HF-O. Principal Coordinate Analysis (PCoA) of UniFrac distances between gut microbial communities of the individuals **(C, E)**, and UniFrac distances between gut microbial communities of the individuals within each group and between the two groups **(D, F)**. Data are presented as mean ± SEM. NS, not significant. ** p < 0.01.

**Fig 4:**
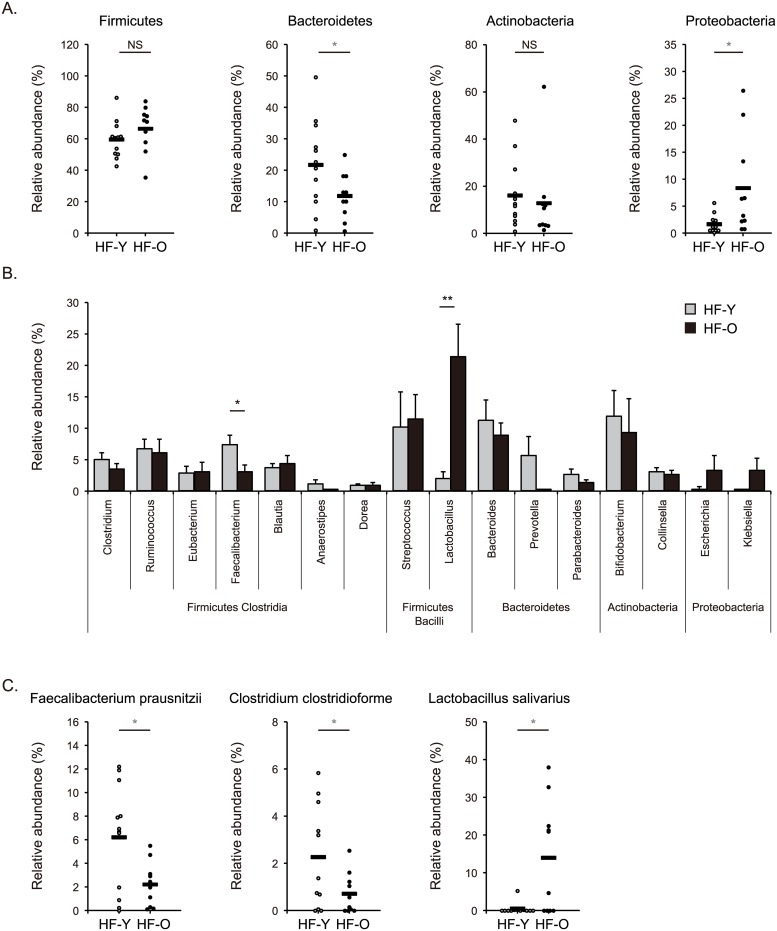
Abundances of taxa in gut microbiota of younger and older patients with heart failure. Relative abundances of taxa in gut microbiota samples obtained from younger and older patients with heart failure (HF-Y and HF-O, respectively). **(A)** Phylum level. **(B)** Genus level. **(C)** Species level. Data are presented as mean ± SEM. Horizontal bars indicate means. * p < 0.05, ** p < 0.01. NS, not significant.

Given that clinical characteristics and outcomes of HF are influenced by aging process [20], we next examined whether gut microbial communities of patients with HF varied according to age. We sequenced 16S rRNA gene amplicons from additional fecal samples obtained from 10 HF patients who were 60 years of age or older. We then compared the composition of gut microbiota of younger HF patients (younger than 60 years of age; n = 12) with that of older HF patients (60 years of age or older; n = 10). The richness and diversity of gut microbial communities within the individual, as evaluated by Chao1-estimated OTU number and Shannon index respectively, were not significantly different between younger and older patients with HF (191 ± 20 vs. 178 ± 13, and 3.38 ± 0.19 vs. 3.21 ± 0.11, respectively) (Fig 3A and 3B). However, both unweighted and weighted UniFrac analyses demonstrated that the differences in gut microbiota composition between the two groups were larger than inter-individual differences in the gut microbiota of older HF group (unweighted UniFrac distance: 0.743 ± 0.004 vs. 0.716 ± 0.007, weighted UniFrac distance: 0.490 ± 0.006 vs. 0.443 ± 0.011; both p < 0.01) (Fig 3C–3F). Interestingly, unweighted but not weighted UniFrac analysis revealed greater inter-individual diversity in the gut microbiota of younger HF patients compared to older patients (Fig 3C–3F). The phylum Bacteroidetes was less abundant (11.7 ± 2.3% vs. 21.7 ± 4.1%, p = 0.047) whereas Proteobacteria was more abundant (8.4 ± 2.9% vs. 1.6 ± 0.5%, p = 0.046) in the gut microbiota of older HF patients than in that of younger patients (Fig 4A). At the genus and species level, the genus *Faecalibacterium*, *F*. *prausnitzii*, and *Clostridium clostridioforme* were reduced (3.1 ± 1.0% vs. 7.5 ± 1.4%, p = 0.021, 2.2 ± 0.6% vs. 6.2 ± 1.3%, p = 0.013, and 0.7 ± 0.3% vs. 2.3 ± 0.6%, p = 0.035, respectively), while the genus *Lactobacillus* and *L*. *salivarius* were enriched (21.4 ± 5.1% vs. 2.0 ± 1.1%, p = 0.004, and 14.0 ± 4.7% vs. 0.4 ± 0.4%, p = 0.018, respectively) in fecal samples from older HF patients compared to younger patients (Fig 4B and 4C).

## Discussion

This study provides the first evidence, to our knowledge, that patients with HF harbor altered gut microbiota, pointing to the potential clinical significance of gut microbiota in the pathophysiology of HF.

In patients with HF, there are disturbances in splanchnic microcirculation because of reduced perfusion, increased congestion, and vasoconstriction caused by neurohumoral activation. These microcirculatory disturbances cause ischemia in the gut, resulting in dysfunction of intestinal epithelial cells [7]. The composition of gut microbiota is influenced by the functional crosstalk with intestinal epithelial cells, for example by antimicrobial proteins secreted from these cells [21]. Previous studies demonstrated that HF patients harbored higher levels of adherent bacteria in the sigmoid mucosal biofilm, as evaluated by fluorescence *in situ* hybridization [5], and larger quantities of pathogenic bacteria in feces such as *Campylobacter*, *Shigella*, and *Salmonella*, as assessed by microbial culture method [22]. In our study, we used high-throughput culture-independent 16S rRNA gene sequencing of fecal samples to analyze the composition of gut microbiota. This technique has revealed the association of gut microbial dysbiosis with many diseases [3, 4], including not only gastrointestinal diseases [23] but also cardiovascular and metabolic diseases such as obesity, diabetes mellitus, and hypertension [24–27]. Our study shows that gut dysbiosis is also associated with HF and that HF-associated gut dysbiosis varies further according to age.

*Eubacterium rectale* and *Dorea longicatena* were reduced in patients with HF, and *Faecalibacterium prausnitzii* and *Clostridium clostridioforme* were less abundant in older HF patients than in younger patients (Figs 2 and 4). *E*. *rectale*, *D*. *longicatena*, and *C*. *clostridioforme* belong to Clostridia cluster XIVa, and *F*. *prausnitzii* is a member of Clostridia cluster IV [28, 29]. *E*. *rectale* and *F*. *prausnitzii* produce the short-chain fatty acid butyrate as a major fermentation product [30]. Butyrate has diverse beneficial effects on the host, such as serving as an energy source for intestinal epithelial cells, regulating epithelial barrier integrity, and suppressing intestinal and extra-intestinal inflammation [31–33]. Diminished proportions of butyrate-producing bacteria in the gut have been associated with several intestinal and extra-intestinal disorders, such as inflammatory bowel diseases, obesity, diabetes mellitus, and hypertension [23–27]. *D*. *longicatena* produces another short-chain fatty acid acetate as a fermentation product [29]. Acetate can be utilized by other microbes to generate butyrate [34]. In contrast, lactate-producing *Lactobacillus* was found to be more abundant in older HF patients than in younger patients. Decreases in acetate- and butyrate-producing bacteria and an increase in lactate-producing bacteria have been demonstrated in animal models of hypertension [25]. It has been reported that germ-free mice have negligible levels of acetate, propionate, and butyrate in plasma and feces, as compared with conventionally-raised mice, indicating that gut microbiota is responsible for generating most of these short-chain fatty acids in the host [35]. We presume that HF-associated gut dysbiosis, which further varies with age, can be characterized by an imbalance in gut microbe-derived metabolites such as short-chain fatty acids. The alteration in gut microbiota and the disruption of gut barrier function may result in aberrant production and absorption of microbe-derived metabolites, which can contribute to cardiac dysfunction, inflammation, and malnutrition in HF patients. Further experiments are required to elucidate the precise effects of gut microbe-derived metabolites on the pathogenesis of HF. While 16S rRNA gene sequencing allows for taxonomic classification of microbes, whole metagenome shotgun sequencing enables the identification of potential metabolic functions of microbiota [36].

Recent studies have demonstrated that HF patients had higher plasma levels of TMAO, a gut microbe-derived metabolite of choline or carnitine, than control subjects, and that elevated plasma TMAO level was associated with higher mortality risk in HF patients [12]. Moreover, increased dietary choline or TMAO intake aggravated adverse cardiac remodeling induced by pressure overload in mice [13]. These observations suggest a potential contribution of TMAO to the development of HF. Several microbial species, such as *Proteus mirabilis*, *Proteus penneri* and *Escherichia fergusonii*, have been identified to be capable of producing trimethylamine (TMA), a precursor of TMAO, *in vitro* as well as in gnotobiotic mouse model [37, 38]. However, none of those species reported were increased in the gut of HF patients in our study. TMA may be produced in the human gut by highly complex community of microbial species both defined and currently undefined to be capable of producing TMA.

The composition of gut microbiota in HF patients can be modified not only by hemodynamic alterations, but also by dietary habits, comorbidities such as hypertension, diabetes, dyslipidemia, and chronic kidney disease, and therapeutic interventions. Medication use has been shown to influence the composition of gut microbiota [39]. Besides antibiotics, several classes of drugs such as proton pump inhibitors (PPIs), statins, β-adrenergic receptor blockers, angiotensin converting enzyme inhibitors, and angiotensin II receptor blockers, have potential effects on the gut microbiome [40]. In particular, PPI use is profoundly associated with gut microbiome composition and function. In PPI users, oral commensal bacteria such as the family Streptococcaceae were increased in the gut, possibly due to the change in acidic environment of the stomach, allowing commensal bacteria in the upper gastrointestinal tract to move beyond to the lower gut [41, 42]. In our study, PPIs were taken by 5 of 12 younger HF patients and by 5 of 10 older HF patients. Although relative abundances of specific oral bacteria were not significantly increased in the gut of HF patients in our study, the observed differences in gut microbiota of HF patients can be affected by confounding factors such as medication. It is challenging to dissect the contribution of each of these factors to the observed differences in gut microbiota of HF patients in the larger-scale studies.

The composition of gut microbiota of older adults is known to be distinct from that of younger adults [43], and gut microbiota profiles of older people vary substantially between individuals, affected by several factors such as diet, habitation, morbidity, and medication [44]. According to a paper that characterized the microbiota composition of 161 subjects aged 65 years and older and 9 younger control subjects in Ireland [43], the microbiota of the elderly was distinct from that of younger subjects, with a greater proportion of the phylum Bacteroidetes and a less proportion of the phylum Firmicutes. Our analysis revealed that the composition of gut microbiota of older HF patients was different from that of younger HF patients (Figs 3 and 4). Interestingly, Bacteroidetes was less abundant in the gut microbiota of older HF patients than in that of younger patients, while Firmicutes was not significantly different (Fig 4A). In addition, the phylum Proteobacteria was more abundant in the gut microbiota of older HF patients (Fig 4A). Therefore, our data implicate that the observed alterations in gut microbiota with aging may occur specifically in HF patients. However, these alterations may be caused by aging-related accumulation of complex background factors such as comorbidities and medications rather than HF. Given the unavailability of gut microbiota profiles of older HC subjects in our study, it is currently uncertain how much HF affects the altered gut microbiota profiles of older HF patients observed. In our study, older HF patients were characterized by higher proportions of hypertension (6 of 10 older patients vs. 1 of 12 younger patients) and HF with preserved ejection fraction (HFpEF) (4 of 10 older patients vs. none of 12 younger patients) (S1 Table). Gut microbiota profiles of older HF patients found in our study were consistent with the previous report on gut microbiome observed in animal models of hypertension in terms of increased lactate-producing bacteria, decreased butyrate-producing bacteria, and diminished proportion of Bacteroidetes [25]. Although pathophysiology of HFpEF remains poorly understood, aging and hypertension are key factors that contribute to pathophysiological process of HFpEF [45]. Gut microbiota alterations observed in older HF patients could have potential effects on the pathophysiology of HFpEF. It remains to be elucidated how gut microbiota changes occurring during aging may affect the host physiological aging process *per se* [46], and further investigations, including analysis of the composition of gut microbiota of elderly HC subjects without comorbidities, will be needed to elucidate the impact of the alterations in microbiota in older HF patients.

Our study cannot provide evidence for direct causal effects. Fecal microbial transplantation studies in animal models of HF harboring human microbiota would be useful to establish the role of gut microbial dysbiosis in the pathophysiology of HF. Finally, this is a cross-sectional study with relatively small size of study population. Large-scale longitudinal studies are required to explore the change in gut microbiome during the course of progression or regression of HF.

Our study reveals a novel link between HF and dysbiosis in gut microbiota. This finding supports the novel concept of heart-gut axis. Further exploration of this axis would lead to breakthroughs in the development of innovative diagnostic and therapeutic approach for HF. Moreover, gut microbiota profiles of HF patients vary significantly between individuals. Personalized characterization of gut microbiome in HF patients could be useful in risk stratification or treatment decision for each individual patient [47].

## Supporting information

S1 Table Clinical characteristics of heart failure patients. (PDF)

S2 Table Clinical characteristics of healthy control subjects. (PDF)

S3 Table Comparison of clinical characteristics of heart failure patients and healthy control subjects. (PDF)
