# Supplementary material for: Dysbiosis and compositional alterations with aging in the gut microbiota of patients with heart failure
Source: PLoS One. 2017 Mar 22;12(3):e0174099. doi: 10.1371/journal.pone.0174099 (PMC5362204; doi:10.1371/journal.pone.0174099)
Supplement: S1 Table — (PDF) [file pone.0174099.s001.pdf]

Supplementary Table S1. Clinical Characteristics of Heart Failure Patients

	Younger HF patients (n = 12)												Older HF patients (n = 10)									
Age, years	26	32	45	47	48	49	49	52	52	54	56	59	61	63	65	67	76	79	79	80	81	87
Male/female	M	M	M	M	M	M	M	M	F	M	M	M	M	M	F	M	M	F	M	M	M	F
BMI, kg/m^2	24.8	18.5	21.3	19.5	29.1	23.0	27.5	16.0	28.8	24.6	20.0	21.3	36.3	23.6	32.9	22.6	24.8	21.3	21.5	18.9	24.1	23.3
Comorbidities																						
Hypertension	No	No	No	No	No	No	Yes	No	No	No	No	No	No	No	Yes	Yes	Yes	Yes	No	No	Yes	Yes
Diabetes mellitus	No	No	No	Yes	No	No	No	No	Yes	No	Yes	Yes	Yes	No	Yes	No	Yes	No	No	No	No	No
Dyslipidemia	No	No	No	Yes	Yes	No	No	No	Yes	No	No	Yes	Yes	No	Yes	No	Yes	Yes	No	No	No	No
CKD	No	No	Yes	No	No	Yes	No	No	Yes	No	No	Yes	Yes	Yes	No	No	Yes	Yes	Yes	Yes	Yes	No
Etiology	DCM	ARVC	VHD	CAD	DCM	HCM	DCM	DCM	DCM	DCM	DCM	CAD	DCM	CAD	CAD	CAD	CAD	HHD	DCM	DCM	HHD	HHD
Ischemic etiology	No	No	No	Yes	No	No	No	No	No	No	No	Yes	No	Yes	Yes	Yes	Yes	No	No	No	No	No
NYHA functional class	III	IV	III	III	II	III	II	II	III	III	IV	III	II	II	II	III	II	III	III	II	III	II
LVEF, %	17	16	16	22	22	17	34	21	15	34	7	19	33	37	34	70	35	75	21	24	50	52
Medications																						
ACE inhibitors or ARBs	Yes	Yes	Yes	Yes	Yes	Yes	Yes	Yes	Yes	No	Yes	Yes	Yes	No	Yes	Yes	Yes	Yes	No	Yes	Yes	Yes
β-blockers	Yes	Yes	Yes	Yes	Yes	Yes	Yes	Yes	Yes	Yes	Yes	Yes	Yes	Yes	Yes	Yes	Yes	Yes	Yes	Yes	Yes	Yes
Loop diuretics	Yes	Yes	Yes	Yes	Yes	Yes	Yes	Yes	Yes	No	Yes	Yes	Yes	Yes	Yes	No	Yes	Yes	Yes	Yes	Yes	No
Statins	No	No	No	Yes	No	No	No	No	No	No	Yes	No	Yes	Yes	Yes	Yes	Yes	Yes	Yes	No	No	No
Aspirin	No	No	No	Yes	No	No	No	No	No	No	No	Yes	No	No	Yes	Yes	Yes	No	No	No	No	No
PPIs	No	No	No	Yes	No	Yes	Yes	Yes	No	No	No	Yes	No	Yes	Yes	No	Yes	No	No	Yes	Yes	No
BNP, pg/ml	526	2150	968	1629	569	1168	432	198	302	1208	2945	632	171	483	152	1012	668	1114	1720	291	1267	99
eGFR, ml/min/1.73m^2	81	60	48	57	52	48	57	57	39	53	61	43	34	23	60	77	19	20	48	28	18	77

ACE = angiotensin converting enzyme; ARB = angiotensin receptor blocker; ARVC = arrhythmogenic right ventricular cardiomyopathy; BMI = body mass index; BNP = B-type natriuretic peptide; CAD = coronary artery disease; CKD = chronic kidney disease; DCM = dilated cardiomyopathy; eGFR = estimated glomerular filtration rate; F = female; HCM = hypertrophic cardiomyopathy; HF = heart failure; HHD = hypertensive heart disease; LVEF = left ventricular ejection fraction assessed by transthoracic echocardiography; M = male; NYHA = New York Heart Association; PPI = proton pump inhibitor; VHD = valvular heart disease
