# Supplementary material for: Dysbiosis and compositional alterations with aging in the gut microbiota of patients with heart failure
Source: PLoS One. 2017 Mar 22;12(3):e0174099. doi: 10.1371/journal.pone.0174099 (PMC5362204; doi:10.1371/journal.pone.0174099)
Supplement: S2 Table — (PDF) [file pone.0174099.s002.pdf]

Supplementary Table S2. Clinical Characteristics of Healthy Control Subjects

	HC subjects (n = 12)											
Age, years	31	34	35	36	37	39	41	45	49	50	50	50
Male/female	M	F	M	F	M	F	M	M	M	M	M	M
BMI, kg/m^2	27.5	23.7	25.0	21.4	23.1	22.6	24.3	21.9	21.0	21.3	25.2	20.9
Comorbidities												
Hypertension	No	No	No	No	No	No	No	No	No	No	No	No
Diabetes mellitus	No	No	No	No	No	No	No	No	No	No	No	No
Dyslipidemia	No	No	No	No	No	No	No	No	No	No	No	No
CKD	No	No	No	No	No	No	No	No	No	No	No	No

BMI = body mass index; CKD = chronic kidney disease; F = female; HC = healthy control; M = male
