# Supplementary material for: Dysbiosis and compositional alterations with aging in the gut microbiota of patients with heart failure
Source: PLoS One. 2017 Mar 22;12(3):e0174099. doi: 10.1371/journal.pone.0174099 (PMC5362204; doi:10.1371/journal.pone.0174099)
Supplement: S3 Table — (PDF) [file pone.0174099.s003.pdf]

Supplementary Table S3. Comparison of Clinical Characteristics of Heart Failure Patients and Healthy Control Subjects

	HC (n = 12)	HF-Y (n = 12)	HF-O (n = 10)	p value (HC vs HF-Y)	p value (HF-Y vs HF-O)
Age, years	41.4 ± 2.0	47.4 ± 2.8	73.8 ± 2.8	0.095	<0.001
Male, %	75	92	70	0.590	0.293
BMI, kg/m^2	23.2 ± 0.6	22.9 ± 1.2	24.9 ± 1.7	0.831	0.339
Comorbidities					
Hypertension, %	0	8	60	1.000	0.020
Diabetes mellitus, %	0	33	30	0.093	1.000
Dyslipidemia, %	0	33	40	0.093	1.000
CKD, %	0	33	70	0.093	0.198
Ischemic etiology, %		17	40		0.348
NYHA functional class		2.9 ± 0.2	2.4 ± 0.2		0.054
LVEF, %		20.0 ± 2.2	43.1 ± 5.8		0.003
Medications					
ACE inhibitors or ARBs, %		92	80		0.571
β-blockers, %		100	100		1.000
Loop diuretics, %		92	80		0.571
Statins, %		17	70		0.027
Aspirin, %		17	30		0.624
PPIs, %		42	50		1.000
BNP, pg/ml		1060.6 ± 238.8	697.7 ± 176.0		0.236
eGFR, ml/min/1.73m^2		54.7 ± 3.1	40.4 ± 7.4		0.102

Data are presented as mean ± SEM or percentage. Continuous variables are compared using unpaired Student *t* test. Frequencies are compared using Fisher's exact test.

ACE = angiotensin converting enzyme; ARB = angiotensin receptor blocker; BMI = body mass index; BNP = B-type natriuretic peptide; CKD = chronic kidney disease; eGFR = estimated glomerular filtration rate; HC = healthy control subjects; HF-O = older heart failure patients; HF-Y = younger heart failure patients; LVEF = left ventricular ejection fraction as assessed by echocardiography; NYHA = New York Heart Association; PPI = proton pump inhibitor
